# Predictor variables of happiness and its connection with risk and protective factors for health

**DOI:** 10.3389/fpsyg.2015.01176

**Published:** 2015-08-12

**Authors:** Maite Garaigordobil

**Affiliations:** Faculty of Psychology, University of the Basque CountryDonostia-San Sebastian, Spain

**Keywords:** psychology, happiness, psychopathology, social behavior, self-esteem, cooperation, sex, adolescence

## Abstract

Great thinkers, philosophers, scientists, and artists from History have often been concerned about one of the most important elements of life: happiness. The study had four goals: (1) To analyze possible differences in feelings of happiness as a function of sex and age; (2) To explore the relations of happiness with risk factors (psychopathological symptoms, behavior problems) and protective factors (self-concept-self-esteem, cooperative behavior, social skills) for health; (3) To identify predictor variables of happiness; and (4) To explore whether self-esteem mediates the relationship between happiness and psychopathological symptoms. The sample comprised 286 adolescents (14–16 years old). The study used a descriptive, correlational, and cross-sectional methodology. Seven assessment instruments were administered. The ANOVAs confirm that there are no sex differences, but happiness decreases as age increases. Pearson coefficients show that adolescents with more feelings of happiness had fewer psychopathological symptoms (somatization, obsession–compulsion, interpersonal sensitivity, depression, anxiety, hostility, phobic anxiety, paranoid ideation, psychoticism…), fewer behavioral problems (school-academic, antisocial behavior, shyness-withdrawal, psychopathological, psychosomatic), high social adaptation, high self-concept/self-esteem, many cooperative behaviors, many appropriate social skills, and few negative social skills (inappropriate assertiveness, impulsiveness, jealousy-withdrawal). Multiple regression analysis identified five variables predicting happiness: high self-concept, few symptoms of depression, many cooperative behaviors, high self-esteem, and low psychoticism. Results showed a partial mediational effect of self-esteem in the relation between happiness and psychopathological symptoms. The discussion focuses on the importance of implementing programs to promote feelings of happiness, as well as protective factors for health (self-esteem, cooperation…).

## Introduction

Great thinkers, philosophers, scientists, and artists from History have often been concerned about one of the most important elements of life: happiness. Although all human beings know and use the concept of happiness, there is no agreement about the definition of this construct. In ancient times, the concept of happiness focused on good luck, and the gods played an important role. Although in recent years, we are witnessing a great interest aroused by this topic, the old time philosophers (Aristotle, Socrates, Epicurus, Seneca…) already argued about it. Aristotle distinguished a moral life (necessary to maintain happiness) from a material life (necessary to meet the basic needs), underlining the need for both (Anderson et al., 2011). For Socrates, virtue is the necessary and sufficient condition for happiness. However, in the *Declaration of Independence* of Jefferson in 1776, the pursuit of happiness was included as a human right. Therefore, happiness went from being related to luck and considered as being passive to being considered a more active construct. Currently, happiness is understood as something that humans beings can control and achieve (Kesebir and Diener, 2008; Oishi, 2012).

In research on happiness, two perspectives are differentiated: hedonism and eudaimonia (Deci and Ryan, 2000; Ryan and Deci, 2001). Hedonic well-being is based on the subjective assessment of quality of life, including both positive and negative affects as well as the cognitive assessment of life satisfaction, and it is related to obtaining pleasure. However, a satisfactory life cannot be associated only with pleasure and therefore, eudaimonic well-being focuses on peoples’ full functioning and commitment; happiness can only be achieved as the result optimal psychological functioning, the consequence of people developing their true nature, their potential, to the full; from the standpoint of eudaimonia, profound well-being requires autonomy, competence, and affiliation or creation of affective links.

Happiness has been defined as the appraisal, both affective and cognitive, of one’s own life, consisting of general satisfaction with life, the presence of positive affects and the absence of negative ones (Diener et al., 1999). Lyubomirsky et al. (2005) define happiness as a subjectively assessed phenomenon that is determined both by positive and negative affects, and by life satisfaction. The RICH theory (Kehle, 1999; Kehle et al., 2002) defines happiness as a synonym of psychological health and, accordingly, happy people have four characteristics: resources (feeling of independence or control over one’s life), intimacy (friendship, empathy, and capacity to enjoy the company of other people), competence (capacities and awareness of these skills), and health (being aware of and practicing healthy behaviors). However, subjective well-being comprehends a broad range of components such as happiness, life satisfaction, hedonic balance, and realization, consisting of the affective and cognitive appraisal of one’s own life (Kim-Prieto et al., 2005).

Regarding sex differences in feelings of happiness, most of the studies carried out with adolescents and adults have found no differences as a function of sex (Huebner et al., 2000; Csikszentmihalyi and Hunter, 2003; Park and Huebner, 2005; Hervás, 2009; Vera et al., 2012; Uusitalo-Malmivaara and Lehto, 2013; Hunagund and Hangal, 2014), but some studies have found significant differences between men and women, with women obtaining higher scores in happiness (Aldous and Ganey, 1999).

With regard to age, few studies have investigated its relation with happiness, and, moreover, the results are discrepant. Some studies have found no differences as a function of age (Huebner et al., 2000; Hervás, 2009), whereas others reveal differences (Lacey et al., 2012). The work of Vera et al. (2012) reported that higher age predicts low levels of happiness, whereas other studies suggest a U-form model, finding that the highest levels of happiness are achieved around 20–29 years of age, and after 50 years (Blanchflower and Oswald, 2006).

The concept of happiness has been studied from different approaches, and diverse investigations have analyzed the correlations of happiness with other constructs such as health, personality traits, behaviors… According to Argyle (1997), happiness affects health and vice versa. When discussing health, we refer both to physical and mental health. Thus, Bartels et al. (2013) found a negative relation between subjective well-being and psychopathology in a sample of adolescents (12–20 years). In the same direction, Agbaria et al. (2012) showed that the happier adolescents are, the fewer symptoms they have. In research with adults, these results are ratified because hedonic behaviors predicted less stress and depression (Henderson et al., 2013).

Another line of research has correlated happiness and personality. In a study carried out with adults, happiness correlated negatively with neuroticism and positively with extraversion (Hills and Argyle, 2001) and openness (Pelechano et al., 2013). In the same direction, Garaigordobil et al. (2009), in a sample aged between 20–40 years, found that subjective psychological well-being was positively related to extraversion and negatively related to neuroticism and psychoticism.

Research with adolescents and university students has underlined that happiness is positively related to friendship (Demir and Davidson, 2013), altruism, social skills (Demir et al., 2012), cooperation (Rigby et al., 1997), academic success, and self-esteem (Salmela-Aro and Tuominen-Soini, 2010; Sato and Yuki, 2014). Recent studies (Cheung et al., 2014) also suggest that people with higher self-control are happier possibly because they are: (1) more promotion-focused on acquiring positive gains thereby facilitating more approach-oriented behaviors, and (2) less prevention-focused on avoiding losses thereby reducing avoidance-oriented behaviors.

Within the debate on the bidirectional relationship between happiness and health, in 2011, the Coca-Cola Institute of Happiness and the Faculty of Psychology at the Complutense University of Madrid (2011), in collaboration with the research team directed by Professor Vazquez of the Complutense University of Madrid, prepared a report entitled “Happiness and the Perception of Health.” In this research, interviews with 3,000 people aged 18–65 were carried out. The results showed that, in the face of a health problem, happier people feel healthier than unhappier people. Health can influence happiness but not all health problems imply a decrease in the level of life satisfaction. In general, the presence of certain psychological problems (depression, stress, insomnia, addictions, anorexia, etc.), physical illnesses, and psychophysiological disorders (cardiovascular diseases, stomachache, migraines/headaches, obesity, cancer, disabilities, etc.) have a negative impact on life satisfaction, whereas other disorders such as hypertension, diabetes, or sexual dysfunction, among others, are not related to life satisfaction. The study also emphasized the relationship between well-being and health, showing that unhappier people have more health problems, with the exception of allergies and skin problems. This relationship was strongest with psychological disorders versus diseases of a more physical nature. In addition, the investigation confirmed the importance of family and friends as a factor that enhances well-being.

Subsequent analyses with data from the sample of this study (Vázquez et al., 2015) have shown that, although physical and psychological problems both had an impact on life satisfaction, greater effect sizes were generally found for psychological rather than for physical illness. Regression analysis, controlling for the effects of comorbidity and sociodemographic variables (e.g., age, gender, marital status, employment status, and educational level), revealed a significant impact on life satisfaction of cancer and migraine (in the domain of physical problems), and of depression, lack of concentration, insomnia, and stress/anxiety (in the domain of psychological problems). Further multivariate analyses were conducted to estimate decreases in the *Satisfaction with Life Scale* for each of the conditions assessed. A lack of significant interactions revealed that the effect of comorbid physical and/or psychological problems could be additive. The present results show that people who have psychological problems display a marked decrease in life satisfaction. On a whole, this negative impact is significantly greater than the decrease associated with physical problems. To see in detail the connections between psychological well-being and health, consult the review of the meta-analytical studies performed by Vázquez (2013).

Regarding variables that predict happiness, in general, few investigations have carried out predictive analyses. Nevertheless, some studies have identified the predictive power of cooperation (Lu and Argyle, 1991) and sociability (Csikszentmihalyi and Hunter, 2003; Easterlin, 2006; Uusitalo-Malmivaara and Lehto, 2013). High levels of self-esteem-self-concept have also been shown to be predictors of psychological well-being (Garaigordobil et al., 2009). Lastly, health has been considered as a factor predicting happiness (Easterlin, 2006; Angner et al., 2013) and the relation between these two variables is bidirectional.

In recent years, research on self-esteem has been gaining relevance within the context of the identification of protective factors against psychological problems. Its importance for personal well-being, mental health, professional success, social relationships, academic performance, and so on has been the focus of numerous research projects in the human and social sciences.

Taking as reference the above-mentioned studies, this work had four goals: (1) To analyze possible differences in feelings of happiness as a function of sex and age; (2) To explore the relations of happiness with risk factors (psychopathological symptoms, behavior problems) and protective factors (self-concept-self-esteem, cooperative behavior, social skills) for health; (3) To identify predictor variables of happiness; and (4) To explore whether self-esteem mediates the relationship between happiness and psychopathological symptoms. Taking into account the relevant role of positive feelings in physical and mental health, and based on the review of previous studies, in this investigation, five hypotheses were proposed: (H1) No sex differences will be found, but, taking into account the typical tensions of adolescence, it is expected that feelings of happiness will decrease between ages 14 and 16 years. (H2) Negative correlations will be found between feelings of happiness and psychopathological symptoms (somatization, obsession–compulsion, interpersonal sensitivity, depression, anxiety, hostility, phobic anxiety, paranoid ideation, psychoticism, and additional) and behavior problems (school-academic problems, antisocial behavior, shyness-withdrawal, psychopathological disorders, anxiety problems, psychosomatic disorders), and negative social skills (inappropriate assertiveness, overconfidence, impulsiveness, jealousy/withdrawal). (H3) Positive correlations will be found between feelings of happiness and protective factors for health, such as self-concept-self-esteem, the capacity of cooperation, and diverse appropriate social skills related to sociability and emotional expression. (H4) High scores in protective factors (many positive social cooperative behaviors, high self-concept-self-esteem) and low scores in risk factors (psychopathological symptoms, behavior problems) will be predictors of feelings of happiness. (H5) Self-esteem will be a mediator variable of the relation between happiness and psychopathological symptoms.

## Materials and Methods

### Participants

The sample comprised 286 Spanish adolescents aged between 14 and 16 years, 148 male (51.7%) and 138 female (48.3%). Of them, 46.5% were 14 years old, 35.2% were 15, and 18.2% were 16 (*M* = 14.72 years, SD = 0.75). The participants belong to six schools of diverse socioeconomic levels (50% public and 50% private). Of the entire sample, 62.9% are in third grade of secondary education and 37.1% are in fourth grade. Of them, 11.9% (*n* = 34) had previously consulted a psychologist for school problems associated with situations of separation from the parents, nervousness-anxiety…, and currently 5.2% (*n* = 15) still have difficulties and are treated by a psychologist. The sample was randomly selected from the list of centers of the province of Gipuzkoa.

### Procedure

The study uses a descriptive and correlational cross-sectional methodology, and attempts to establish concomitant relations among feelings of happiness and a broad range of variables (psychopathological symptoms, behavior problems, self-concept-self-esteem, capacity of cooperation, and social skills), as well as to identify predictor variables. For this purpose, firstly, a letter was sent to the headmasters of the randomly selected schools, explaining the project and requesting their collaboration. Those who agreed to collaborate were interviewed in order to present the project and give them the informed consent forms for the participants’ parents. If the center director decided not to collaborate, the procedure was repeated with the next center on the list, taking into account the network (private–public) and/or the socio-economic-cultural level of the center that would not participate. After receiving the parents’ consent, a team of psychology graduates went to the schools to administer the assessment instruments. The adolescents completed the six assessment instruments in two 30-min sessions. In addition, they received an envelope containing a scale of behavior problems (BPS) to be completed by their parents. This study met the ethical values required in research with human beings, respecting the fundamental principles (informed consent and right to information, protection of personal data and guarantees of confidentiality, non-discrimination, and freedom to leave the study at any stage). This study was carried out in accordance with the recommendations of the Vice-Rectorate of Research of the University of the Basque Country (UPV/EHU). This Vice-Rectorate approved the study and partially financed it (1/UPV0006.231-H-15910.2004).

### Instruments

Seven assessment instruments with psychometric guarantees were used to measure the following variables: happiness, psychopathological symptoms, behavioral problems, self-concept, self-esteem, capacity of cooperation, and social skills.

#### The Oxford Happiness Questionnaire (OHQ; Hills and Argyle, 2002)

The OHQ was derived from the *Oxford Happiness Inventory* (OHI), which, reduced 29 items, attempts to measure the happiness of a general nature of each individual, that is, psychological well-being. For example, “I am not particularly optimistic about the future,” “I am well satisfied about everything in my life,” “I am very happy,” “Life is good,” and “I always have a cheerful effect on others”… The person expresses the degree of agreement with the statements on a 6-point Likert scale (1 = strongly disagree; 6 = strongly agree). The studies carried out with a sample of people aged between 13 and 68 years verified the good reliability of this scale (α = 0.91). The construct validity of the OHI was assessed through its associations with different measures of individual differences of cognitive traits and variables. In the original study, the associations of the OHI and the OHQ were compared, obtaining significant correlations. In this sample, Cronbach’s α reached 0.86, indicating good internal consistency.

#### Symptoms Checklist-90-Revised (SCL-90-R; Derogatis, 1983; Spanish adaptation of González de Rivera et al., 2002)

This self-report has 90 items distributed into 10 scales, which report the psychopathological disorders: *somatization* (bodily dysfunctions, neurovegetative disorders in cardiovascular, respiratory, gastrointestinal, and muscular systems), *obsession–compulsion* (behaviors, thoughts, and impulses that the subject considers absurd and undesirable, which create deep anguish and which are hard to resist, avoid, or get rid of), *interpersonal sensitivity* (feelings of shyness and embarrassment, tendency to feel inferior to others, hypersensitivity to other people’s opinions and attitudes and, in general, awkwardness and inhibition in interpersonal relations), *depression* (clinical signs and symptoms of depressive disorders, including dysphoric experiences, anhedonia, hopelessness, impotence, and lack of energy, as well as self-destructive ideas and other cognitive and somatic manifestations typical of depressive states), *anxiety* (clinical manifestations of anxiety, both generalized and acute or “panic,” including general signs of emotional stress and its psychosomatic manifestations), *hostility* (thoughts, feelings and behaviors of aggressiveness, anger, irritability, rage, and resentment), *phobic anxiety* (focusing more on the scale of social phobia and agoraphobia symptoms than on that of simple phobia), *paranoid ideation* (paranoid behavior, considered mainly as the response to a delusional disorder including suspiciousness, self-referential centralism and delirious ideation, hostility, grandiosity, fear of loss of autonomy, and need for control), *psychoticism* (in the general population, it is more frequently associated with feelings of social alienation than with clinically manifest psychosis), and *additional scale* (miscellaneous symptoms that make up a clear referent of melancholic depression). Furthermore, the test makes it possible to calculate the *General Symptomatic Index* (GSI), which is a standard and indiscriminate measure of the intensity of global psychosomatic and psychic suffering, the *Positive Symptom Total* (PST), which is the number of existing symptoms, and the *Positive Symptom Distress Index* (PSDI), which links suffering or global distress with the number of symptoms. Adolescents report the frequency with which they have experienced these symptoms during the last month. Studies have shown the reliability of the test (α = between 0.81 and 0.90) and construct validity. The internal consistency obtained with the sample of this study was suitable for the whole set of symptoms (GSI, α = 0.89).

#### Behavioral Problems Scale (BPS; Navarro et al., 1993)

This 99-item scale is filled in by the parents to assess behavioral problems. The items are grouped in seven scales: *school-academic problems* (related to low academic performance), *antisocial behavior* (behaviors that may be classified as aggressive, and other behaviors that are not aggressive but might impair social relationships), *shyness-withdrawal* (tendency to solitude and susceptibility in social relationships), *psychopathological disorders* (serious problems which generally have a depressive component), *anxiety problems* (behaviors that express fear and/or anxiety generalized form), *psychosomatic disorders* (physical disorders without medical cause), and a positive scale of *social adjustment* (adjustment to social rules). Parents must report whether or not their children engage in these behaviors. As regards the reliability of the scale, information about the internal consistency of the whole BPS has been gathered (α = 0.88). To test the criterion validity, the BPS was applied to different samples of children and adolescents (referred by the school psychologist due to problems at school, referred to a clinical psychologist, and prison inmates due to criminal problems), and the multiple regression analysis showed that belonging to different criterion groups was the variable that presented the highest level of relations with the BPS scores. Reliability analysis with the sample of this study showed good internal consistency (α = between 0.70 and 0.83).

#### Adjective Checklist for Self-Concept Assessment with Adolescents and Adults (ACSA; Garaigordobil, 2011)

This checklist is made up of 57 positive adjectives (attractive, friendly, cooperative, intelligent, creative…) which respondents are asked to score on a scale of 0–4 (*not at all – very much*) according to the degree to which the items define or describe their personality. Studies of validity and reliability confirm its psychometric properties. A study carried out with a sample of 1,578 participants obtained a Cronbach’s alpha of 0.92. In order to analyze the validity of the ACSA, correlations were calculated with other instrument measuring self-esteem (RSE; Rosenberg, 1965), obtaining significant positive correlations (*r* = 0.63, *p* < 0.001). Furthermore, other analysis carried out indicated significant negative relationships of self-concept (ACSA) with psychopathological symptoms (Derogatis, 1983). Reliability analysis with the sample of this study presented high internal consistency (Cronbach’s alpha = 0.95).

#### Self-Esteem Scale (RSE; Rosenberg, 1965)

This scale assesses general self-esteem with 10 statements focusing on global feelings of self-appraisal (“On the whole, I am satisfied with myself”); five of them are drafted positively the other five negatively. The subject must read the statements and report the extent to which they apply to him/her, using a Likert-type scale with four response categories (ranging from *strongly agree* to *strongly disagree*). The reliability of the test has been broadly documented in the literature. McCarthy and Hoge (1982) have reported consistency coefficients (Cronbach’s α) ranging from 0.74 to 0.77, and test–retest reliability of 0.63 (interval of 7 months) and of 0.85 (interval of 2 weeks). The validity of the scale as a one-dimensional measure of self-esteem has also been proven in several studies (Silber and Tippett, 1965). The internal consistency obtained with the sample of the present study was adequate (α = 0.82).

#### Cooperativeness Scale (CS; Rigby et al., 1997)

This 18-item scale measures the individual’s capacity to cooperate with others, defining cooperation as behaving conjointly and coordinately at work, leisure, or in social relationships, for the pleasure of sharing activities, goals, or simply to enhance relationships. Participants rate their responses on a 5-point Likert scale, ranging from 1 (*totally disagree*) to 5 (*totally agree*). Of the 18 items of the test, nine reflect cooperative attitudes and nine uncooperative attitudes. Item examples are: “Team work is the best way to get results” “It is more productive to work alone.” In the authors’ study, conducted in Australia with a sample of adolescents, adequate reliability was obtained (Cronbach’s α = 0.77). In this same study, concurrent validity was examined in a sample of students, controlling age and obtaining correlations with other measures. The results showed that the correlation of cooperation with the number of friends was low (*r* < 0.20), and the quality of friendships was a better indicator of cooperation than the number of friends, thus confirming a significant link between cooperation and happiness. Reliability analysis with the sample of this study showed good internal consistency (α = 0.87).

#### The Matson Evaluation of Social Skills in Youngsters (MESSY; Matson et al., 1983; Spanish adaptation of Méndez et al., 2002)

The scale evaluates five factors: *Appropriate Social Skills* (behaviors such as emotional expressiveness, having friends, sharing); *Inappropriate Assertiveness* (aggressive behaviors, making fun of or abusing others); *Impulsiveness* (behaviors such as getting angry easily or interrupting others); *Overconfidence* (overvaluing oneself); and *Jealousy-Withdrawal* (feelings of loneliness, lack of friends). Item examples of each scale are: “I know how to make friends” (social skills), “I get back at people who offend me” (inappropriate assertiveness), “I interrupt others when they are talking” (impulsiveness), “I like to brag about the things I have” (overconfidence), and “I feel jealous of others” (jealousy/withdrawal). This instrument is made up of 62 items, with response options rated on a 4-point Likert scale ranging from 1 (*never*) to 4 (*always*). A study carried out with the Spanish version of the MESSY in a sample of 634 adolescents of ages 12–17 showed high internal consistency (Cronbach’s α = 0.88). In this study, Pearson correlation coefficients were calculated between the total MESSY score and other measures of assertiveness and social skills. Inappropriate social behaviors had negative correlations with prosocial behavior and positive correlations with aggressiveness and antisocial behavior. The internal consistency obtained with the sample of the present study was adequate in appropriate skills (α = 0.86) and negative behaviors (α = 0.84).

## Results

### Feelings of Happiness: Differences as a Function of Sex and Age

In order to analyze possible differences in feelings of happiness as a function of sex and age, after confirming the basic assumptions (homogeneity, homoscedasticity…), we performed analysis of variance, the results of which are presented in **Table 1**.

**Table 1:** Means and SD in feelings of happiness by sex and age.

Sex	Age	*n*	*M*	SD
Male	14	61	129.82	15.45
	15	52	118.08	19.67
	16	35	117.49	17.68
	Total	148	122.78	18.41
Female	14	72	122.99	20.10
	15	49	120.39	17.30
	16	17	115.12	18.86
	Total	138	121.09	19.04
Total	14	133	126.12	18.37
	15	101	119.20	18.50
	16	52	116.71	17.92
	Total	286	121.97	18.70

The results (see **Table 1**) show that there were no differences between boys and girls in feelings of happiness, *F*(1,284) = 0.09, *p* = 0.343, η^2^ = 0.003; *r* = 0.05. However, there were significant differences as a function of age, *F*(2,283) = 7.20, *p* = 0.001; η^2^ = 0.049; *r* = 0.22, observing a decrease in happiness as age increased from 14 to 16 years. In addition, the Bonferroni group comparison tests showed that the group of 14-year-olds scored significantly higher than the groups of 15- and 16-year-olds (*Post hoc*: 14 > 15,16). The Sex^∗^Age interaction was not significant, *F*(1,284) = 1.79, *p* = 0.168, η^2^ = 0.013; *r* = 0.11.

### Feelings of Happiness: Relations with Psychopathological Symptoms, Behavior Problems, Self-Concept-Self-Esteem, Cooperative Behavior, and Social Skills

To explore the relations between feelings of happiness, risk factors (psychopathological symptoms, behavior problems), and protective factors (cooperation, social skills, self-concept-self-esteem) for health, we performed Pearson correlation analyses with the entire sample, the results of which are presented in **Table 2**.

**Table 2:** Pearson correlation coefficients between happiness and psychopathology, behavioral problems, self-concept, self-esteem, and social behaviors.

	Happiness
**SCL-90-R. Psychopathological Symptoms**	
Somatization	-0.33^∗∗∗^
Obsession–compulsion	-0.46^∗∗∗^
Interpersonal sensitivity	-0.55^∗∗∗^
Depression	-0.58^∗∗∗^
Anxiety	-0.40^∗∗∗^
Hostility	-0.43^∗∗∗^
Phobic anxiety	-0.37^∗∗∗^
Paranoid ideation	-0.42^∗∗∗^
Psychoticism	-0.52^∗∗∗^
Additional	-0.47^∗∗∗^
General Symptomatic Index (GSI)	-0.58^∗∗∗^
Positive Symptom Total (PST)	-0.55^∗∗∗^
Positive Symptom Distress Index (PSDI)	-0.54^∗∗∗^
**BPS. Behavioral Problems**	
School-academic problems	-0.32^∗∗∗^
Antisocial behavior	-0.21^∗∗^
Shyness-withdrawal	-0.29^∗∗∗^
Psychopathological disorders	-0.24^∗∗∗^
Anxiety problems	-0.06 ns
Psychosomatic disorders	-0.30^∗∗∗^
Social adaptation	0.36^∗∗∗^
Behavioral problems total	-0.35^∗∗∗^
**ACSA – RSE. Self-concept/self-esteem**	
Self-concept	-0.61^∗∗∗^
Self-esteem	-0.54^∗∗∗^
**CS. Cooperativeness**	
Cooperative behavior	0.47^∗∗∗^
**MESSY. Social skills**	
Appropriate social skills	0.33^∗∗∗^
Inappropriate assertiveness	-0.27^∗∗∗^
Impulsiveness	-0.32^∗∗∗^
Overconfidence	-0.12^∗^
Jealousy-withdrawal	-0.52^∗∗∗^
Negative social skills total	-0.38^∗∗∗^

*^∗^*p* < 0.05, ^∗∗^*p* < 0.01, ^∗∗∗^*p* < 0.001, ns = non-significant.*

The results (see **Table 2**) confirmed, firstly, significant correlations between feelings of happiness and all the psychopathological symptoms assessed, which were of a greater magnitude (moderate-large) with symptoms of interpersonal sensitivity, depression, psychoticism, and the three global psychopathology indexes. Therefore, the data suggest that adolescents with considerable feelings of happiness had fewer psychopathological symptoms of somatization, obsession–compulsion, interpersonal sensitivity, depression, anxiety, hostility, phobic anxiety, paranoid ideation, psychoticism, additional, and lower levels in the indexes GSI, PST, and PSDI.

Secondly, negative correlations (of a medium-low magnitude) were also found between happiness and parent-assessed behavior problems (school-academic problems, antisocial behavior, shyness-withdrawal, psychopathological disorders, psychosomatic disorders), and positive correlations with social adjustment. No significant relation was found with anxiety problems. The data show that adolescents with high scores in happiness had few school-academic problems, infrequent antisocial behavior, few problems of shyness-withdrawal, few psychopathological and psychosomatic disorders, as well as high social adjustment (see **Table 2**).

Thirdly, positive correlations between feelings of happiness and self-concept/self-esteem, capacity for cooperation and appropriate social skills were found, as well as negative correlations with negative social skills (inappropriate assertiveness, impulsiveness, overconfidence, jealousy-withdrawal). The relations with self-concept/self-esteem were of a large magnitude, and the relations with cooperative behavior and jealousy-withdrawal were moderate. Hence, the data show that adolescents with high scores in happiness had high self-concept, high self-esteem, they performed many cooperative behaviors with others, and had appropriate social skills related to sociability and emotional expression (see **Table 2**).

In order to contrast the results obtained with the correlations observed, we explored whether the adolescents who had high scores in feelings of happiness displayed significant differences in the target variables of the study when comparing them with adolescents who obtained low or medium scores. For this purpose, firstly, we divided the sample into three profiles as a function of their scores on the OHQ, emphasizing the extremes: Profile 1 (low level of happiness, percentile scores 1–15; 16.8%), Profile 2 (medium level of happiness, percentile scores 16–84; 67.8%) and Profile 3 (high level of happiness percentiles ≤85; 15.4%). Subsequently, we performed descriptive analyses (means and SDs) and analysis of variance as a function of the happiness profile, calculating the effect size (Eta), and *post hoc* group comparisons (Bonferroni), the results of which are presented in **Table 3**.

**Table 3:** Means and SD of all the variables in the three happiness profiles (low, medium, high), and results of the analysis of variance as a function of profile, effect size (Eta), and *post hoc* tests (Bonferroni).

	Profile 1 (*n* = 48)	Profile 2 (*n* = 194)	Profile 3 (*n* = 44)	*F*(2,284) profile	Eta	*Post hoc*
	*M* (SD)	*M* (SD)	*M* (SD)			
**SCL-90-R. Psychopathological Symptoms**					
Somatization	1.22 (0.72)	0.74 (0.54)	0.57 (0.38)	7.87^∗∗∗^	0.113	1 > 2,3
Obsession–compulsion	1.51 (0.75)	0.86 (0.64)	0.53 (0.31)	13.67^∗∗∗^	0.181	1 > 2,3
Interpersonal sensitivity	1.67 (0.84)	0.82 (0.58)	0.48 (0.28)	22.49^∗∗∗^	0.266	1 > 2,3
Depression	1.75 (1.01)	0.63 (0.51)	0.30 (0.20)	37.34^∗∗∗^	0.376	1 > 2 > 3
Anxiety	1.01 (0.84)	0.47 (0.42)	0.35 (0.34)	11.32^∗∗∗^	0.154	1 > 2,3
Hostility	1.40 (1.12)	0.51 (0.49)	0.29 (0.26)	21.35^∗∗∗^	0.256	1 > 2,3
Phobic anxiety	0.57 (0.67)	0.18 (0.29)	0.09 (0.14)	11.26^∗∗∗^	0.154	1 > 2,3
Paranoid ideation	1.27 (0.71)	0.64 (0.59)	0.40 (0.30)	13.03^∗∗∗^	0.174	1 > 2,3
Psychoticism	0.91 (0.81)	0.27 (0.36)	0.06 (0.10)	22.13^∗∗∗^	0.263	1 > 2,3
Additional	1.46 (0.93)	0.71 (0.57)	0.36 (0.34)	17.88^∗∗∗^	0.224	1 > 2 > 3
GSI	1.31 (0.68)	0.60 (0.38)	0.35 (0.17)	30.59^∗∗∗^	0.330	1 > 2 > 3
PST	54.79 (18.94)	35.53 (17.08)	25.74 (11.16)	16.73^∗∗∗^	0.213	1 > 2 > 3
PSDI	2.03 (0.54)	1.44 (0.38)	1.23 (0.16)	25.57^∗∗∗^	0.292	1 > 2,3
**BPS. Behavioral Problems**					
School-academic problems	11.79 (5.21)	5.88 (5.71)	3.91 (5.64)	11.32^∗∗∗^	0.154	1 > 2,3
Antisocial behavior	10.58 (7.27)	5.29 (7.27)	4.78 (3.50)	11.66^∗∗∗^	0.158	1 > 2,3
Shyness-withdrawal	9.37 (4.93)	6.80 (3.77)	6.00 (3.39)	4.36^∗^	0.066	1 > 2,3
Psychopathological disorders	5.63 (4.42)	3.01 (2.47)	2.87 (2.94)	6.68^∗∗^	0.097	1 > 2,3
Anxiety problems	5.42 (2.16)	4.72 (2.72)	4.22 (2.43)	1.12 ns	0.018	–
Psychosomatic disorders	3.42 (2.56)	1.67 (2.32)	1.22 (1.16)	6.04^∗∗^	0.089	1 > 2,3
Social adaptation	20.84 (4.29)	25.34 (4.15)	26.65 (3.79)	11.83^∗∗∗^	0.160	1 > 2,3
Behavioral problems total	46.21 (17.44)	27.38 (15.10)	23.00 (13.48)	14.42^∗∗∗^	0.189	1 > 2,3
**ACSA – RSE. Self-concept/self-esteem**					
Self-concept	2.05 (0.41)	2.56 (0.44)	2.90 (0.26)	22.64^∗∗∗^	0.268	1 < 2 < 3
Self-esteem	24.53 (5.62)	30.11 (5.20)	34.04 (2.94)	19.36^∗∗∗^	0.238	1 < 2 < 3
**CS. Cooperativeness**					
Cooperative behavior	64.58 (8.12)	69.69 (7.58)	79.26 (4.27)	23.95^∗∗∗^	0.279	1 < 2 < 3
**MESSY. Social skills**					
Appropriate social skills	66.84 (7.81)	70.81 (8.08)	74.26 (7.65)	4.51^∗^	0.068	1 < 3
Inappropriate assertiveness	29.42 (7.77)	25.48 (6.21)	23.22 (4.69)	5.26^∗^	0.078	1 > 2,3
Impulsiveness	10.84 (1.89)	9.48 (2.21)	8.52 (1.56)	6.58^∗∗^	0.096	1 > 2,3
Overconfidence	8.11 (2.92)	8.24 (2.31)	7.87 (1.54)	0.23 ns	0.004	–
Jealousy-withdrawal	14.21 (4.03)	10.68 (2.82)	9.17 (1.07)	17.59^∗∗∗^	0.221	1 > 2,3
Negative social skills total	62.58 (1.07)	53.88 (10.83)	48.78 (7.27)	8.82^∗∗∗^	0.125	1 > 2,3

*^∗^*p* < 0.05, ^∗∗^*p* < 0.01, ^∗∗∗^*p* < 0.001, ns, non-significant. Profile 1 low happiness (percentile 1–15). Profile 2 medium happiness (percentile 16–84). Profile 3 high happiness (≤85); Eta, Effect size; *post hoc* = Bonferroni group comparison test.*

As shown in **Table 3**, the adolescents who had low scores in feelings of happiness (Profile 1), had significantly more psychopathological symptoms (in all the scales assessed and in the three global indexes), and many behavior problems (school-academic, antisocial behavior, shyness-withdrawal, psychopathological and psychosomatic problems). Moreover, the adolescents with few feelings of happiness had low self-concept/self-esteem, few cooperative behaviors, few appropriate social skills, and many negative social skills (inappropriate assertiveness, impulsiveness, jealousy-withdrawal). However, no differences in anxiety problems or overconfidence were found among the three happiness profiles. The Bonferroni tests confirmed that the adolescents included in Profile 1 (low level of happiness) had significant differences with those of Profiles 2 and 3 (medium and high level of happiness).

### Feelings of Happiness: Predictive Variables

To identify the variables that predict a high score in feelings of happiness, we performed stepwise multiple linear regression analysis, including all the variables, the results of which are presented in **Table 4**.

**Table 4:** Multiple regression analysis for predictor variables of happiness.

	*R*	*R*^2^	Δ*R*^2^	*B*	SE	Constant	β	*t*	*p*
ACSA self-concept	0.658	0.433	0.429	11.57	2.66	55.19	0.284	4.33	0.000
SCL-90-R depression	0.765	0.585	0.578	-5.75	2.40	82.82	-0.215	-2.39	0.018
CS cooperative behavior	0.815	0.664	0.656	0.66	0.13	45.95	0.287	4.81	0.000
RSE self-esteem	0.822	0.676	0.665	0.56	0.24	35.21	0.165	2.30	0.023
SCL-90-R psychoticism	0.829	0.688	0.675	-6.49	2.99	36.73	-0.168	-2.16	0.032

Out of all the predictor variables of feelings of happiness among adolescents (see **Table 4**), five were statistically significant: self-concept (β = 0.284), depression (β = -0.215), cooperative behaviors (β = 0.287), self-esteem (β = 0.165), and psychoticism (β = -2.16). The standardized Beta regression coefficients indicate that these variables have a relevant impact on the variable “feelings of happiness.” According to this, the percentages of explained variance (adjusted determination coefficients) for each one of these predictor variables were of an important magnitude in all the variables (42.9, 57.8, 65.6, 66.5, and 67.5%). Five variables explain 67.5% of the variance and are predictors of “happiness”: high self-concept, few symptoms of depression, many cooperative behaviors, high self-esteem, and low psychoticism.

### Self-Esteem as a Mediator between Happiness and Psychopathological Symptoms

To analyze whether self-esteem is mediator between happiness and psychopathological symptoms (GSI), we performed linear regression analysis. The results showed a partial mediation of self-esteem in the inverse relation between happiness and psychopathological symptoms (total effect β = -1.47, *p* = 0.001; partial effect, β = -1.14, *p* = 0.000; Sobel test, *Z* = -4.53, *p* = 0.000). This result shows a partial mediational effect of self-esteem in the inverse relation between happiness and psychopathological symptoms. That is, even though the happiness is low, if the adolescent’s self-esteem is high, such low happiness does not predict many psychopathological symptoms.

## Discussion

The most important objectives of the study were to explore feelings happiness as a function of sex and age, to analyze its relations with risk and protective factors for health, identifying variables that predict feelings of happiness. Firstly, the results confirmed that there were no sex differences in feelings of happiness, but such feelings decrease as age increases from 14 to 16 years. These results confirm our Hypothesis 1, pointing in the same direction as other studies that have not found any sex differences (Huebner et al., 2000; Csikszentmihalyi and Hunter, 2003; Park and Huebner, 2005; Hervás, 2009; Vera et al., 2012; Uusitalo-Malmivaara and Lehto, 2013; Hunagund and Hangal, 2014). However, they contradict studies finding that women score higher than men in happiness (Aldous and Ganey, 1999). The absence of sex differences can be interpreted from the hypothesis of progressive homogeneity between males and females that is emerging in recent studies. In addition, the results confirm studies finding differences as a function of age (Vera et al., 2012), but they disagree with those that have found no differences (Huebner et al., 2000; Hervás, 2009). The decrease in happiness at these ages may be explained by the increase of tensions and anxiety that occur during adolescence. The discrepancies with other studies may be related to the diverse ages of the samples and to the different instruments employed.

Secondly, the results show that the adolescents with high scores in happiness had fewer psychopathological symptoms (somatization, obsession–compulsion, interpersonal sensitivity, depression, anxiety, hostility, phobic anxiety, paranoid ideation, psychoticism, and additional, GSI, PSDI, PST), fewer behavior problems (school-academic problems, antisocial behavior, shyness-withdrawal, psychopathological, and psychosomatic disorders), and few negative social skills (inappropriate assertiveness, impulsiveness, jealousy-withdrawal). These results confirm Hypothesis 2 almost completely (no relations were found with anxiety problems) and point in the same direction as other studies finding fewer psychopathological symptoms in happy adolescents (Agbaria et al., 2012; Bartels et al., 2013) as well as greater academic success (Salmela-Aro and Tuominen-Soini, 2010).

Thirdly, the results suggest that adolescents with high feelings of happiness also have high self-concept-self-esteem, perform many cooperative behaviors, display high social adjustment and many appropriate social skills related to sociability emotional expression. These results confirm Hypothesis 3 and are consistent with other studies underlining that happiness is positively related to friendship (Demir and Davidson, 2013), cooperation (Rigby et al., 1997), altruism, social skills (Demir et al., 2012), and self-esteem (Salmela-Aro and Tuominen-Soini, 2010; Sato and Yuki, 2014).

Lastly, the study identifies five variables predicting feelings of happiness, such as high self-concept, few symptoms of depression, many cooperative behaviors, high self-esteem, and low psychoticism. These data confirm Hypothesis 4, which postulated that high scores in protective factors (many positive social cooperative behaviors, high self-concept/self-esteem) and low scores in risk factors (psychopathological symptoms and behavior problems) would be predictor variables. These results confirm the theoretical models that have considered sociability as a factor of happiness, as well as the studies identifying the predictive power of the capacity to cooperate (Lu and Argyle, 1991), social relations (Easterlin, 2006; Uusitalo-Malmivaara and Lehto, 2013), self-concept and self-esteem (Garaigordobil et al., 2009). Complementarily, the results found a partial mediational effect of self-esteem in the relation between happiness and psychopathological symptoms, that confirm the Hypothesis 5.

Taking into account the studies that have shown the important role of positive feelings for physical and mental health, the results of the present study represent a significant contribution to our knowledge. The work provides evidence of the connections of happiness with lower levels of psychopathological symptoms, and behavior problems, with high levels of self-concept-self-esteem, cooperative behavior, appropriate social skills and few negative social behaviors. Moreover, the work has allowed us to identify that a high level of self-esteem-self-concept, many cooperative behaviors and few symptoms of depression and psychoticism (social alienation) predict happiness, which is useful for the design of intervention programs to promote positive feelings of happiness. The strong point of the study is having assessed psychopathological symptoms both with self-reports and parent-reports. Other significant contribution of the study was to reveal the mediator role (protective factor) of self-esteem in the connection between happiness and psychopathological symptoms.

Thus, the results of the study suggest that intervention programs that promote positive feelings of happiness, as well as protective factors for health, such as self-concept-self-esteem, cooperation, and social skills, can play a relevant role in the reduction of psychopathological symptoms and behavior problems (antisocial behavior, academic problems, psychosomatic symptoms). As limitations of the study, we mention the small age range of the sample of this study (14–16 years) and the fact that it is a cross-sectional study.

The meeting held by the United Nations (2011) pointed out that the increase in chronic diseases represents a worldwide crisis. The accumulative cost of these diseases in the next the 20 years (2011–2030) will reach 30.4 trillion dollars (Bloom et al., 2011), 46.7 trillion dollars if incurable mental diseases are also counted. This sum would be much lower if intervention programs were implemented to promote positive emotions and feelings of happiness, which have shown that happiness and health are related. That is, the implementation of socio-emotional intervention programs would prevent mental health problems, thereby saving thousands of dollars and increasing the population’s happiness.

In spite of being the most important life goal, the pursuit of happiness has been considered by many a frivolity and an utopia. Can one pursue happiness? Can one learn from happiness? (see Avia, 2008). Independently of the circumstances (promoting or hindering happiness), not everyone is equally able to feel happiness. The capacity to enjoy life depends on many factors, among others, biological characteristics and personality traits, which are consolidated and configured throughout childhood… Therefore, we should be aware that the programs aimed at fostering well-being and happiness have their limitations. The team of Lyubomirski et al. (2005) has pointed out that such programs can stimulate an increase in happiness of approximately 40%, whereas personal circumstances can add 10% to the baseline of satisfaction of each person. Intervention programs can help but they cannot change a person completely, although they can modify the cognitive and motivational processes so that circumstances are reinterpreted, processed, or experienced in a positive way, which may be related to resilience to adversity. To a great extent, happiness is more a state of mind than something conditioned by circumstances.

That said, after reviewing some studies analyzing happiness, several behavioral and cognitive strategies are noted that promote feelings of happiness and that can be taken into account when designing intervention programs to foster such feelings, for example: (1) remain active and physically occupied (a healthy mind in a healthy body); (2) share activities with other people and do things for others; (3) focus on the present, preventing past experiences or concerns about the future from distorting the “here and now,” because happiness is an internal emotional state that can only be felt in the present; (4) set small goals to be sequenced toward a larger goal; (5) think positive, have positive thoughts; (6) set feasible goals; (7) be capable of enjoying pleasant things, paying close attention and slowly savoring the things that cause pleasure; (8) learn to give oneself small daily rewards; (9) accept what cannot be changed, learning to forgive and be reconciled with the past, no matter how negative it may have been; (10) practice self-compassion to forgive oneself and feel thankful to others, because one’s own happiness is related to that of others; (11) be aware of the positive aspects of life and feel thankful for them; (12) learn new things and cultivate appreciation of beauty and excellence (a picture, a sculpture, a musical melody, a sunset…); (13) coherence between cognition, emotion, and behavior (what one thinks, feels, and does); (14) develop resilience or the capacity to cope with adversity and overcome it without letting it destroy or hurt one; and (15) love and be loved, have feelings of love for others and feel loved by the people around one.

## Conflict of Interest Statement

The author declares that the research was conducted in the absence of any commercial or financial relationships that could be construed as a potential conflict of interest.
